# Supplementary material for: miRNAs as Biomolecular Markers for Food Safety, Quality, and Traceability in Poultry Meat—A Preliminary Study
Source: Molecules. 2024 Feb 6;29(4):748. doi: 10.3390/molecules29040748 (PMC10891583; doi:10.3390/molecules29040748)
Supplement: Supplementary file 1 [file molecules-29-00748-s001.zip › molecules-2811833-supplementary.pdf]

Supplementary Materials:

1. **Table S1**. Expression of 2 housekeeping genes (U6 and 5S) in the muscle (A), lung (B), spleen (C) tissues for the two breeds (R308 and RG).

2. **Table S2**: Relative expression of miR-21 and miR-126 in muscle, lung and spleen tissues for R308 and RG.

1. Table S1.

A. *Expression of 2 housekeeping genes (U6 and 5S) in the muscle tissues for the two breeds (R308 and RG).*

Muscle Ct are average values of 3 technical replicates. The experiment was conducted twice.

U6 snRNA			
		Ct Average	SD
R308	cDNA 1	27,72	0,17
R308	cDNA 2	26,90	0,04
R308	cDNA 3	29,50	0,14
R308	cDNA 4	28,51	0,28
R308	cDNA 5	29,29	0,17
R308	cDNA 6	27,22	0,09
RG	cDNA 7	29,13	0,12
RG	cDNA 8	27,65	0,15
RG	cDNA 9	27,85	0,16
RG	cDNA 10	26,70	0,08
RG	cDNA 11	28,33	0,06

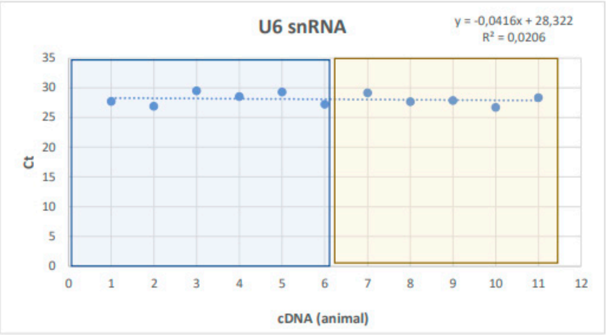

5S rRNA			
		Ct Average	SD
R308	cDNA 1	20,91	0,13
R308	cDNA 2	19,65	0,03
R308	cDNA 3	20,33	0,10
R308	cDNA 4	19,72	0,13
R308	cDNA 5	20,77	0,15
R308	cDNA 6	19,52	0,05
RG	cDNA 7	21,02	0,06
RG	cDNA 8	20,82	0,09
RG	cDNA 9	20,96	0,01
RG	cDNA 10	19,97	0,08
RG	cDNA 11	20,17	0,09

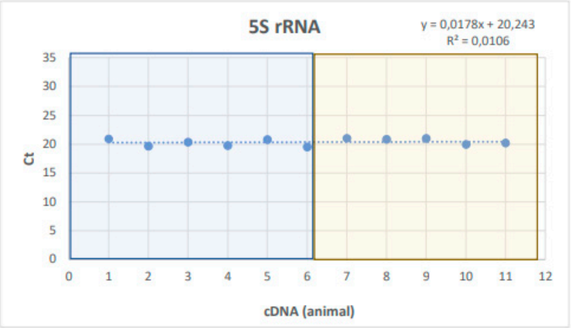

B. *Expression of 2 housekeeping genes (U6 and 5S) in the lung tissues for the two breeds (R308 and RG).*

Lung Ct are average values of 3 technical replicates. The experiment was conducted twice.

U6 snRNA			
		Ct Average	SD
R308	cDNA 1	19,59	0,02
R308	cDNA 2	20,19	0,04
R308	cDNA 3	21,35	0,05
R308	cDNA 4	20,25	0,08
R308	cDNA 5	20,66	0,03
R308	cDNA 6	20,57	0,13
RG	cDNA 7	21,03	0,10
RG	cDNA 8	21,17	0,09
RG	cDNA 9	21,92	0,04
RG	cDNA 10	20,60	0,09
RG	cDNA 11	20,93	0,10

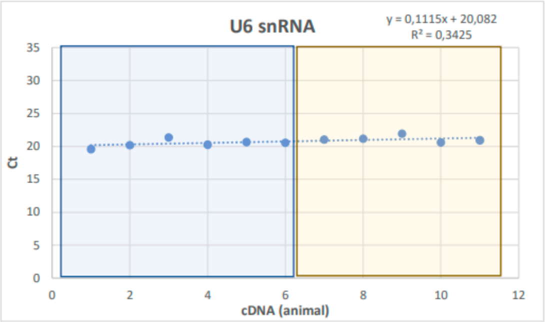

5S rRNA			
		Ct Average	SD
R308	cDNA 1	18,82	0,21
R308	cDNA 2	19,77	0,14
R308	cDNA 3	18,83	0,01
R308	cDNA 4	20,33	0,07
R308	cDNA 5	20,46	0,21
R308	cDNA 6	19,66	0,03
RG	cDNA 7	19,91	0,12
RG	cDNA 8	20,50	0,20
RG	cDNA 9	20,67	0,14
RG	cDNA 10	19,75	0,09
RG	cDNA 11	19,78	0,03

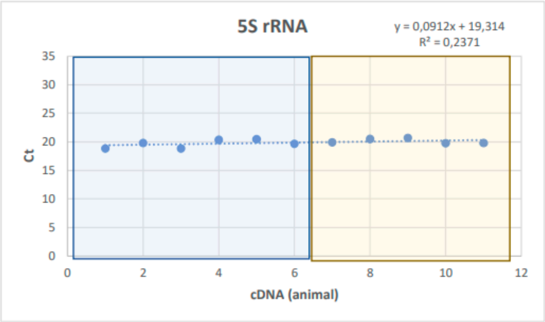

C. *Expression of 2 housekeeping genes (U6 and 5S) in the spleen tissues for the two breeds (R308 and RG).*

Spleen Ct are average values of 3 technical replicates. The experiment was conducted twice.

U6 snRNA			
		Ct Average	SD
R308	cDNA 1	14,93	0,14
R308	cDNA 2	14,70	0,03
R308	cDNA 3	15,59	0,02
R308	cDNA 4	14,70	0,20
R308	cDNA 5	15,83	0,25
R308	cDNA 6	15,33	0,11
RG	cDNA 7	15,80	0,09
RG	cDNA 8	15,27	0,11
RG	cDNA 9	15,31	0,05
RG	cDNA 10	15,26	0,05
RG	cDNA 11	14,97	0,11

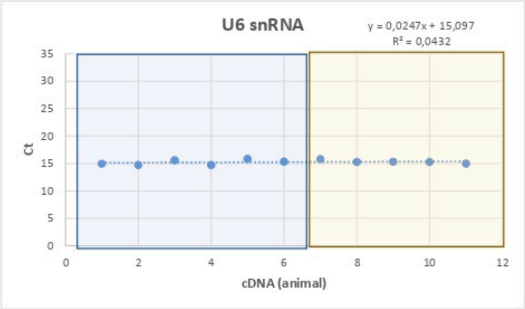

5S rRNA			
		Ct Average	SD
R308	cDNA 1	19,96	0,21
R308	cDNA 2	18,95	0,05
R308	cDNA 3	18,98	0,11
R308	cDNA 4	18,70	0,13
R308	cDNA 5	20,58	0,26
R308	cDNA 6	18,90	0,11
RG	cDNA 7	19,88	0,04
RG	cDNA 8	20,50	0,28
RG	cDNA 9	19,92	0,17
RG	cDNA 10	18,98	0,09
RG	cDNA 11	19,85	0,18

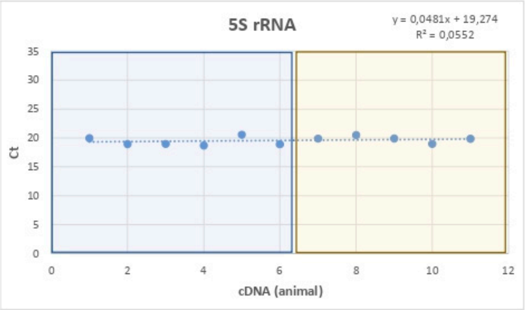

2. Table S2.

**miR-21**	**R308**	**RG**
Tissue	*RE (FC)*	*RE (FC)*
Muscle	1,746	0,504
Lung	1,176	1,952
Spleen	1,498	1,227
**miR-126**	**R308**	**RG**
Tissue	*RE (FC)*	*RE (FC)*
Muscle	1,3	1,133
Lung	1,025 **	3,024 **
Spleen	2,079	1,594

RE, relative expression to internal control (see methods). FC, fold change.
